# Hip Structural Analysis and Multiple Fragility Fractures: Evidence from a Large Cross-Sectional Clinical Cohort

**DOI:** 10.1007/s00223-026-01625-4

**Published:** 2026-10-18

**Authors:** Hamzah Amin, Ajmal Elhani, Amaan Shaffi, Marwan Bukhari

**Affiliations:** 1 Faculty of Health and Medicine, Lancaster University Medical School, Lancaster University, Sir John Fisher Drive, LA1 4YW, Lancaster, UK; 2 Department of Rheumatology, University Hospitals of Morecambe Bay NHS Foundation Trust, Lancaster, UK

## Abstract

Hip structural analysis parameters (HSAP) are associated with hip fracture risk, but associations with multiple fragility fracture phenotypes remain poorly characterised. We analysed 11 DXA-derived HSAP in patients referred between June 2004 and November 2019. Five mutually exclusive fracture phenotypes were defined: no fracture, single hip fracture, single non-hip fracture, multiple non-hip fractures, and multiple fractures including at least one hip fracture. Separate multinomial logistic regression models were fitted for each HSAP reported as odds ratio (OR) per standard deviation increase, adjusted for FRAX covariates and left femoral BMD. Benjamini-Hochberg false discovery rate correction was applied across all tests. A right-hip sensitivity analysis assessed bilateral consistency. 35,647 patients were included (mean age 64.5 years, 82% female). Of 13,777 (38.6%) with a fragility fracture, 1044 (2.9%) had a single hip fracture, 9723 (27.3%) a single non-hip fracture, 2268 (6.4%) multiple non-hip fractures, and 742 (2.1%) multiple fractures including at least one hip fracture. After multiple testing correction, no meaningful associations were observed for isolated hip or non-hip fracture phenotypes. Multiple fractures including at least one hip fracture were independently associated with greater hip axis length (OR 1.44, 95% CI 1.28 to 1.63), neck diameter (OR 1.24, 95% CI 1.11 to 1.38), and centre of mass to upper neck margin distance (OR 1.35, 95% CI 1.21 to 1.51). HSAP were significantly associated with multiple fractures including at least one hip fracture rather than isolated fracture phenotypes, potentially reflecting local structural vulnerability and broader biomechanical instability.

**Supplementary Information:**

The online version contains supplementary material available at https://doi.org/10.1007/s00223-026-01625-4.

## Introduction

Osteoporosis is characterised by reduced bone mineral density (BMD) and compromised microarchitecture [1, 2], predisposing individuals to low-impact fragility fractures, defined as fractures occurring from standing height or less, that carry increased morbidity and mortality [3, 4]. Fragility fractures are estimated to cost the United Kingdom NHS £4.6 bn per year, a burden expected to rise as the population ages [5]. Because osteoporosis remains asymptomatic until a fracture occurs, clinical management focuses on identifying patients at high risk of fragility fractures before they occur, so that antiresorptive therapy can be started in time [6]. Clinically, patients at high risk of fragility fractures are identified using fracture risk assessment tools such as FRAX and QFracture, which are now standard in clinical practice [7]. FRAX, for example, incorporates traditional risk factors for fragility fracture, including age, sex, body mass index (BMI) and bone mineral density, and comorbidities such as rheumatoid arthritis [7]. More recently, an updated version of FRAX, currently in development, is set to incorporate additional predictors of fracture [8]. One such predictor is hip axis length (HAL), a key hip structural analysis (HSA) variable, whose inclusion reflects a growing recognition of the importance of skeletal structural factors beyond BMD [9].

HSA provides DXA-derived geometric measures of the proximal femur, including HAL, neck-shaft angle (NSA), femoral neck width, cross-sectional area (CSA), cortical thickness (CT), section modulus (Z), and buckling ratio (BR) [10, 11]. Large cohort studies and meta-analyses have shown that wider femoral necks, longer HAL, and higher BR independently raise the risk of incident hip fracture, beyond BMD alone [12–14]. In one meta-analysis of 11 studies, greater HAL (OR 1.53, 95% CI 1.06–2.21), femoral neck angle (OR 1.47, 95% CI 1.01–2.15), and neck width (OR 2.68, 95% CI 1.84–3.91) were all significantly associated with femoral neck fracture risk [15]. Similarly, the Women’s Health Initiative (WHI) cohort (*n* ≈ 10,300) found intertrochanteric outer diameter and buckling ratio independently predicted hip fracture (HR ≈ 1.43–1.61 per SD) [13].

While most research has focused on the association between HSA parameters and a single fracture event (usually hip fracture) [12–14], it is plausible that structural fragility at the hip also predisposes individuals to multiple fragility fractures [16]. Subsequent fracture risk is substantial: within a year of an initial fracture, the risk of a further fracture has been reported to reach as high as 32% [17], representing a clinically significant fracture cascade. Previous research into predictors of multiple fragility fractures has highlighted falls risk and alcohol consumption as an important contributing factor, suggesting that biomechanical stability may play a key role in determining fracture susceptibility [18]. In this context, HSA parameters, which reflect proximal femoral geometry, have also been linked to biomechanical instability [19] and may offer additional insight into the relationship between biomechanical instability and the risk of multiple fragility fractures, an area that remains underexplored and forms the rationale for the current study.

### Aim

The aim of this study is to investigate the association between HSA parameters and the odds of experiencing multiple fragility fractures in a large observational clinical cohort.

## Methods

### Data Collection

Patients were referred to our regional dual-energy X-ray absorptiometry (DXA) service in northwest England between 2004 and 2024. At the time of scanning, all patients underwent a standardised clinical assessment to document recognised fragility fracture risk factors, including variables incorporated within FRAX. These included age, sex, body mass index (BMI), rheumatoid arthritis, current glucocorticoid therapy (> 5 mg prednisolone daily for > 3 months), current smoking status, excess alcohol consumption (≥ 3 units daily), and family history of fracture. Height and weight were recorded at the time of attendance. A detailed fracture history was also obtained, including prior fragility fractures and anatomical site (for example hip, wrist, humerus, tibia, and vertebra). All data was stored in a SQL server where it was subsequently extracted and cleaned ready for analysis.

### DXA Scanning Procedure

Between June 2004 and November 2019, the service operated a GE Lunar Prodigy system configured to generate HSA outputs. Following subsequent hardware replacements (2019 – present), HSA outputs were no longer routinely available. Hence, we restricted our analysis to patients who underwent a scan between 2004 and 2019. The DXA scanner in operation undergoes weekly quality control using scanning phantoms to ensure accuracy of measurements.

DXA scanning was performed in accordance with International Society for Clinical Densitometry guidance [20]. This included obtaining bilateral femoral and lumbar spine views as standard where possible [20]. When the femoral region was scanned this produced bone density data at the femoral head, neck, Ward’s region, trochanteric region, and proximal shaft, with BMD recorded at each site. In addition, HSA variables were available for both hips, including hip axis length, strength index, cross-sectional moment of inertia, cross-sectional area, geometric neck distances (d1–d3), neck-shaft angle, centroid position, and buckling ratio. Buckling ratio was calculated by the investigators using the methodology described by Zhang and defined as the ratio of femoral neck outer radius to cortical thickness, with higher values indicating greater cortical instability [21].

### Statistical Analysis

Although clinical data were available from 2004 to 2024, analyses were restricted to patients scanned between 2004 and November 2019 because HSA outputs were only available during this interval. Bilateral HSA measurements were available; however, the primary analysis was restricted to the left hip given it is the non-dominant hip and often has lower bone density when compared to the right due to reduced mechanical loading [22]. Additionally, it is a relatively common practice in HSA studies to also utilise the left hip in statistical models [23].

Baseline FRAX and HSA variables were first compared between patients reporting no fracture a single fragility fracture and those reporting multiple fragility fractures. Continuous variables were analysed using a one-way welch ANOVA test, assuming normality given the large sample size, while categorical variables were compared using Pearson’s chi-squared test.

While the primary aim of this study was to investigate how HSA parameters are associated with multiple fragility fractures, a single dichotomisation in our modelling would not allow us to appreciate the true relationship. Given this, fracture status was classified into five mutually exclusive phenotypes: no fracture, single hip fracture, single non-hip fracture, multiple non-hip fractures, and multiple fractures including at least one hip fracture. All multinomial models used the no fracture category as the reference unless stated otherwise. Each model included one HSA parameter entered separately and was adjusted for age at scan, sex, BMI, current glucocorticoid use, rheumatoid arthritis, excess alcohol consumption (≥ 3 units daily), current smoking, family history of fracture, and left total femoral BMD. We opted to add in each HSA parameter in a separate model to avoid issues with collinearity, with odds ratio being reported per standard deviation increase for each HSA parameter. Furthermore to account for multiple testing across 11 HSA parameters and five multinomial contrasts per model, p-values were adjusted using the Benjamini–Hochberg false discovery rate procedure [24]. All models underwent appropriate model diagnostics including the variance inflation factor to assess for collinearity.

A sensitivity analysis was conducted to perform the same analysis we conducted as above but replaced HSA parameters with values from the right hip. This was to investigate whether there was bilateral consistency in the results. An additional supplementary analysis reported the unadjusted versus adjusted results for our results across both the left and right hip.

## Results

A total of 35,647 patients were included in the study cohort, with a mean age of 64.53 years (SD 12.90) and mean BMI of 27.09 kg/m² (SD 5.58); 29,101 (81.6%) were female. Fracture status was classified into three mutually exclusive groups for baseline comparisons: no fracture (*n* = 21,870, 61.4%), single fracture (*n* = 10,767, 30.2%), and multiple fractures (*n* = 3,010, 8.4%). Among those with fractures, 1,044 had a single hip fracture, 9723 a single non-hip fracture, 2268 multiple non-hip fractures, and 742 multiple fractures including at least one hip fracture.

Across the three groups, significant differences were observed for age, BMI, hip axis length, strength index, femoral head-to-minimum CSMI section distance, cross-sectional moment of inertia, cross-sectional area, centre of mass-to-upper neck margin distance, buckling ratio, and left total femoral BMD (all *p* ≤ 0.03). In general, increasing fracture burden was associated with lower femoral strength and structural measures, including strength index, cross-sectional moment of inertia, cross-sectional area, and BMD, while buckling ratio increased across groups (all *p* < 0.001). Significant differences were also observed for female sex, smoking, excess alcohol use, glucocorticoid use, rheumatoid arthritis, family history of fracture, and hip fracture (all *p* < 0.001). The prevalence of hip fracture increased markedly with fracture burden, from 9.7% among patients with a single fracture to 24.7% among those with multiple fractures. No significant differences were observed for femoral head-to-shaft axis distance (*p* = 0.36), neck diameter (*p* = 0.24), neck-shaft angle (*p* = 0.66), or femoral shaft angle (*p* = 0.63*). Full baseline demographic, clinical, BMD and HSA comparisons are shown in Table 1.

**Table 1:** Comparison of baseline demographics including HSA parameters versus those reporting a single fragility fracture and those reporting multiple fragility fracture

Variable	Overall (n=35,647)	No fracture (n=21,870)	Single fracture (n=10,767)	Multiple fractures (n=3010)	*P*-value
Age at scan (years)	64.53 (12.90)	63.15 (13.27)	66.72 (12.02)	66.75 (11.77)	<0.001
BMI (kg/m^2^)	27.09 (5.58)	27.13 (5.56)	27.09 (5.61)	26.85 (5.53)	0.03
Hip axis length (mm)	108.54 (8.92)	108.64 (9.13)	108.44 (8.65)	108.14 (8.26)	0.01
Strength index	1.44 (0.39)	1.46 (0.39)	1.41 (0.39)	1.38 (0.38)	<0.001
Femoral head to Min-CSMI section (mm)	16.75 (3.52)	16.87 (3.50)	16.57 (3.56)	16.48 (3.54)	<0.001
Femoral head to shaft axis (mm)	49.41 (6.48)	49.44 (6.49)	49.39 (6.47)	49.25 (6.43)	0.36
Neck diameter (mm)	33.57 (3.15)	33.56 (3.22)	33.61 (3.04)	33.50 (3.06)	0.24
Neck-shaft angle (°)	125.80 (5.84)	125.78 (5.82)	125.84 (5.87)	125.79 (5.84)	0.66
Cross-sectional moment of inertia (mm^4^)	10,174.07 (3549.89)	10,400.05 (3646.32)	9875.39 (3383.08)	9508.02 (3206.25)	<0.001
Cross-sectional area (mm^2^)	133.42 (27.00)	136.87 (27.41)	128.63 (25.53)	123.98 (23.98)	<0.001
Centre of mass to upper neck margin (mm)	17.31 (1.93)	17.26 (1.97)	17.40 (1.87)	17.40 (1.88)	<0.001
Femoral shaft angle (°)	2.14 (1.72)	2.16 (1.71)	2.12 (1.74)	2.10 (1.74)	0.63
Buckling ratio	13.15 (2.79)	12.79 (2.71)	13.67 (2.81)	14.07 (2.79)	<0.001
Left total femoral BMD (g/cm^2^)	0.90 (0.16)	0.93 (0.16)	0.87 (0.15)	0.83 (0.14)	<0.001
Female sex, n (%)	29,101 (81.6%)	17,505 (80.0%)	8983 (83.4%)	2613 (86.8%)	<0.001
Current smoker, n (%)	4133 (11.6%)	2671 (12.2%)	1169 (10.9%)	293 (9.7%)	<0.001
Excess alcohol use, n (%)	2149 (6.0%)	1211 (5.5%)	702 (6.5%)	236 (7.8%)	<0.001
Current glucocorticoid use, n (%)	4248 (11.9%)	3113 (14.2%)	883 (8.2%)	252 (8.4%)	<0.001
Rheumatoid arthritis, n (%)	2434 (6.8%)	1606 (7.3%)	631 (5.9%)	197 (6.5%)	<0.001
Family history of fracture, n (%)	7350 (20.6%)	4484 (20.5%)	2109 (19.6%)	757 (25.1%)	<0.001
Hip fracture, n (%)	1786 (5.0%)	0 (0.0%)	1044 (9.7%)	742 (24.7%)	<0.001

SD = standard deviation. Overall = entire cohort (no fracture + single + multiple fracture). *P*-values from one-way Welch ANOVA (continuous) or Pearson’s chi-squared test (categorical), compared jointly across all three groups.

### Left Hip HSA and Multiple Fragility Fractures

When comparing fracture phenotypes against the no-fracture reference group, no HSA parameters were significantly associated with single hip fracture, single non-hip fracture, or multiple non-hip fracture phenotypes following Benjamini–Hochberg false discovery rate correction. In contrast, in the multiple fracture including at least one hip fracture group, several geometric parameters remained significantly associated following correction, including Hip Axis Length (OR 1.44, 95% CI 1.28–1.63), Neck Diameter (OR 1.24, 95% CI 1.11–1.38), Cross-Sectional Moment of Inertia (OR 1.25, 95% CI 1.09–1.42), and Centre of Mass to Upper Neck Margin distance (OR 1.35, 95% CI 1.21–1.51) all per 1 standard deviation increase. Interestingly, there were no significant associations when comparing patients with a single hip fracture with those with multiple fractures including at least one hip fracture. Full results are presented in Table 2.

**Table 2:** Multinomial logistic regression evaluating the association between left side HSA parameters and varying fracture phenotypes after adjusting for confounders

HSA Parameter	Single hip vs. No Fx	Single non-hip vs. No Fx	Multiple non-hip vs. No Fx	Multiple incl. hip vs. No Fx	Multiple incl. hip vs. single hip
Hip axis length (mm)	1.50 (1.07–2.09)	1.04 (0.82–1.31)	1.06 (0.49–2.30)	1.44 (1.28–1.63)***	0.94 (0.80–1.11)
Strength index	0.87 (0.61–1.24)	1.02 (0.80–1.29)	1.00 (0.44–2.29)	0.86 (0.77–0.97)	1.00 (0.86–1.16)
Femoral head to min-CSMI section (mm)	1.08 (0.78–1.51)	0.99 (0.79–1.25)	0.97 (0.45–2.09)	1.06 (0.97–1.16)	0.97 (0.85–1.10)
Femoral head to shaft axis (mm)	1.04 (0.75–1.45)	1.04 (0.83–1.31)	1.08 (0.50–2.34)	1.09 (0.99–1.20)	1.04 (0.91–1.19)
Neck diameter (mm)	1.31 (0.94–1.82)	1.01 (0.80–1.28)	1.01 (0.47–2.17)	1.24 (1.11–1.38)**	0.95 (0.81–1.12)
Neck-shaft angle (°)	1.01 (0.73–1.41)	0.98 (0.78–1.24)	0.93 (0.43–2.01)	1.05 (0.96–1.15)	1.04 (0.91–1.18)
Cross-sectional moment of inertia (mm⁴)	1.18 (0.85–1.65)	1.03 (0.81–1.29)	1.05 (0.47–2.36)	1.25 (1.09–1.42)*	1.06 (0.88–1.27)
Cross-sectional area (mm²)	1.12 (0.80–1.56)	1.03 (0.82–1.30)	1.07 (0.36–3.13)	1.18 (0.99–1.40)	1.06 (0.85–1.33)
Centre of mass to upper neck margin (mm)	1.41 (1.01–1.97)	1.02 (0.81–1.28)	1.04 (0.48–2.24)	1.35 (1.21–1.51)***	0.96 (0.82–1.12)
Femoral shaft angle (°)	1.13 (0.60–2.13)	0.93 (0.55–1.59)	0.88 (0.16–4.89)	1.08 (0.89–1.31)	0.98 (0.72–1.33)
Buckling ratio	1.34 (0.96–1.87)	0.99 (0.78–1.25)	0.93 (0.30–2.89)	1.14 (1.00–1.31)	0.83 (0.68–1.03)

Values: OR per 1 SD increase in HSA parameter (95% CI). Asterisks = FDR-adjusted significance (* *p* < 0.05, ** *p* < 0.01, *** *p* < 0.001).

Columns 1–4: multinomial logistic regression, reference = no fracture. Column 5: binary logistic regression, reference = single hip fracture.

BH-FDR correction applied jointly across all 55 tests (44 multinomial + 11 binary). One adjusted model per HSA parameter.

Red = increased association, Green = decreased association.

### Sensitivity Analysis

When analysing right hip HSA parameters against the no-fracture reference group, no HSA parameters were significantly associated with single hip fracture, single non-hip fracture, or multiple non-hip fracture phenotypes following Benjamini–Hochberg false discovery rate correction. In the multiple fracture group including at least one hip fracture, several right hip geometric parameters remained significantly associated with fracture phenotype, demonstrating a pattern broadly similar to the left hip. These included Hip Axis Length (OR 1.37, 95% CI 1.22–1.54), Strength Index (OR 0.82, 95% CI 0.73–0.92), Neck Diameter (OR 1.20, 95% CI 1.07–1.33), and Centre of Mass to Upper Neck Margin distance (OR 1.30, 95% CI 1.17–1.45), all of which remained significant following false discovery rate adjustment and are interpreted as OR per 1 standard deviation increase. Notably, hip axis length, neck diameter, and centre of mass to upper neck margin distance were also significant in the left hip analysis, supporting bilateral consistency of these associations, while the right hip additionally demonstrated a significant association with lower Strength Index. Overall, the right hip demonstrated a closely comparable pattern to the left hip, with significant associations concentrated in the more severe fracture phenotype involving hip fracture. These results are presented in Table 3.

**Table 3:** Multinomial logistic regression evaluating the association between right side HSA parameters and varying fracture phenotypes

HSA parameter	Single hip vs. No Fx	Single non-hip vs. No Fx	Multiple non-hip vs. No Fx	Multiple incl. hip vs. No Fx	Multiple incl. hip vs. Single Hip
Hip axis length (mm)	1.54 (1.04–2.27)	1.04 (0.83–1.31)	1.06 (0.50–2.25)	1.37 (1.22–1.54)***	0.88 (0.75–1.05)
Strength index	0.82 (0.55–1.23)	1.01 (0.80–1.27)	1.01 (0.45–2.28)	0.82 (0.73–0.92)*	1.00 (0.86–1.15)
Femoral head to min-CSMI section (mm)	1.14 (0.77–1.68)	0.97 (0.77–1.22)	0.97 (0.46–2.03)	1.04 (0.95–1.14)	0.90 (0.79–1.02)
Femoral head to shaft axis (mm)	1.05 (0.71–1.55)	1.05 (0.84–1.32)	1.08 (0.51–2.30)	1.07 (0.97–1.17)	1.01 (0.89–1.16)
Neck diameter (mm)	1.33 (0.90–1.96)	1.00 (0.80–1.26)	1.00 (0.47–2.11)	1.20 (1.07–1.33)*	0.88 (0.75–1.03)
Neck-shaft angle (°)	0.99 (0.67–1.46)	0.97 (0.77–1.23)	0.97 (0.46–2.05)	1.02 (0.93–1.12)	1.06 (0.93–1.20)
Cross-sectional moment of inertia (mm⁴)	1.27 (0.86–1.87)	1.01 (0.80–1.27)	1.04 (0.47–2.31)	1.14 (0.99–1.30)	0.89 (0.74–1.06)
Cross-sectional area (mm²)	1.21 (0.82–1.78)	1.02 (0.81–1.29)	1.03 (0.35–2.98)	1.09 (0.92–1.31)	0.90 (0.72–1.11)
Centre of mass to upper neck margin (mm)	1.55 (1.05–2.29)	1.03 (0.82–1.30)	1.03 (0.48–2.20)	1.30 (1.17–1.45)***	0.83 (0.71–0.97)
Femoral shaft angle (°)	1.24 (0.58–2.63)	0.99 (0.58–1.68)	0.97 (0.21–4.54)	1.04 (0.86–1.26)	0.81 (0.59–1.08)
Buckling ratio	1.32 (0.90–1.95)	0.97 (0.77–1.22)	0.93 (0.30–2.86)	1.18 (1.03–1.35)	0.88 (0.72–1.08)

Values: OR per 1 SD increase in HSA parameter (95% CI). Asterisks = FDR-adjusted significance (*p < 0.05, **p < 0.01, ***p< 0.001).

Columns 1–4: multinomial logistic regression, reference = no fracture. Column 5: binary logistic regression, reference = single hip fracture.

BH-FDR correction applied jointly across all 55 right-hip tests (44 multinomial + 11 binary). One adjusted model per HSA parameter.

Red = increased association, Green = decreased association.

### Supplementary Analyses

When looking at adjusted and unadjusted models in our supplementary analysis we found that many HSA parameters were associated with single hip, single non-hip, and multiple non hip fractures in unadjusted models. However, once FRAX related variables were adjusted for these association were no longer significant. However as per our primary and sensitivity analyses we found that some HSA parameters were associated with multiple fragility fractures including at least one hip, even after FRAX covariate adjustment, with relative bilateral consistency. Our analyses looking at adjusted vs. unadjusted models can be found in the supplementary file.

## Discussion

To our knowledge, this is one of the largest single-centre studies examining DXA-derived HSA parameters across distinct fragility fracture phenotypes. In a cohort of 35,647 patients scanned over 15 years, we applied a five-category fracture classification using multinomial logistic regression, adjusting for conventional FRAX risk factors and total femoral BMD. The principal finding was that several HSA parameters were significantly associated with multiple fragility fractures including at least one hip fracture, the most severe fracture phenotype examined.

The phenotype specificity of these associations is notable. Although isolated hip fractures were more common than multiple fractures including a hip fracture (*n* = 1044 vs. *n* = 742), significant associations with HSA parameters were concentrated in the latter group. This suggests that proximal femoral geometry may be more strongly associated with a severe fracture phenotype characterised by multiple fractures than with isolated hip fracture alone. However, our cross-sectional design prevents assessment of the temporal sequence of fractures within the multiple-fracture group. Previous longitudinal studies have shown that an initial fragility fracture at one skeletal site is associated with increased risk of subsequent fractures at other sites, including vertebral, hip and humeral fractures [16, 25, 26]. Whether HSA characteristics are associated with particular index fracture sites or instead represent markers of underlying susceptibility to a broader fracture phenotype, remains uncertain. Prospective studies incorporating the timing and anatomical site of individual fractures are therefore required to determine whether proximal femoral geometry has a role in predicting fracture cascades. This may be clinically relevant given that refracture rates within one year may reach 32% and fracture risk increases following subsequent fracture events [17].

An important negative finding was the absence of significant associations between HSA parameters and multiple fractures including a hip fracture when compared directly with single hip fracture. This contrasts with the associations observed when compared with individuals without fracture. The smaller numbers in these groups may have limited statistical power; alternatively, following a hip fracture, subsequent fracture risk may be driven more strongly by factors beyond proximal femoral geometry, including skeletal fragility, falls, functional status and treatment exposure [27]. Importantly, this does not negate the primary associations, which were observed consistently at both hips, and suggests that HSA may be most useful for identifying individuals with a more severe fracture phenotype compared with those without fracture. Furthermore, although many HSA parameters were associated with fracture phenotypes in our supplementary unadjusted analyses, these associations were attenuated after adjustment, suggesting that their effects may be largely captured by established FRAX variables.

The HSA parameters most strongly associated with multiple fractures including a hip fracture were centre of mass to upper neck margin distance, femoral neck diameter and hip axis length. Each reflects an aspect of proximal femoral architecture that may influence mechanical response to loading. A greater centre of mass to upper neck margin distance may indicate inferior displacement of the bone centroid, potentially increasing tensile stress in the superior cortex during a sideways fall [28]. Wider femoral necks may be accompanied by proportionally thinner cortices when expansion is not matched by structural reinforcement [29], while greater hip axis length may increase bending forces transmitted through the femoral neck during impact [14]. Collectively, these findings support a role for proximal femoral geometry in mechanical susceptibility to fracture. However, the presence of non-hip fractures within the multiple-fracture phenotype raises the possibility that these associations may not be explained solely by local hip biomechanics.

One possible explanation is that proximal femoral geometry may correlate with other characteristics relevant to fracture susceptibility. Hip structure has been associated with local BMD and tissue composition, including lean mass [30], while adverse gynoid fat distribution has been associated with increased fall risk [31]. Higher levels of adiposity at the hips have also been associated with both hip and non-hip fragility fractures [32]. Although our study cannot establish these relationships mechanistically, these findings raise the possibility that the combination of bone density, local tissue composition and proximal femoral geometry may contribute to more complex fracture phenotypes. Future studies integrating HSA with local body composition, BMD and measures of falls or physical function could help determine whether these factors interact to identify individuals at particularly high risk of multiple fractures.

Our findings are broadly consistent with previous studies showing that greater hip axis length and wider femoral neck geometry are associated with hip fracture [12–14], but extend the existing literature in two important respects. First, the strongest associations in our study were concentrated in multiple fractures including a hip fracture, rather than in the isolated hip fracture group, a clinically relevant phenotype that has not been specifically examined previously. Previous meta-analyses and individual studies [12–14] have generally considered hip fracture as a single outcome, whereas our more granular classification suggests that these associations may be particularly relevant to individuals with a more extensive fracture history. Second, not all HSA parameters remained independently significant after multivariable adjustment, suggesting that some associations may overlap with BMD or established FRAX risk factors. These findings raise the possibility that a limited number of HSA parameters could contribute to future fracture prediction models aimed at identifying individuals at risk of multiple fragility fractures, although this requires validation in prospective cohorts.

### Strengths and Limitations

The strengths of this study include its large sample size, long study period and adjustment for multiple established fracture risk factors, including BMD. Several limitations should also be considered. This was an observational, cross-sectional study, predominantly involving female White patients referred to a single specialist service, which may limit generalisability and introduce referral or confounding-by-indication bias. We were also unable to establish the chronological sequence of fractures or determine whether HSA characteristics preceded or followed individual fracture events. Fractures were patient-reported and may therefore have been subject to recall or reporting bias. Finally, the relatively small number of patients in some fracture phenotypes may have reduced statistical power for between-phenotype comparisons. Additionally, while efforts were made to account for the effect of gender by adjusting for this in our models, stratifying analyses by gender may have been more useful though not possible in the current study due to sample size and multiple comparison considerations. We must also acknowledge that we did not have reliable information on anti-osteoporosis treatment and this has the potential to influence out findings.

## Conclusions

HSA parameters were associated with multiple fragility fractures including at least one hip fracture, the most severe fracture phenotype we examined. Greater hip axis length, femoral neck diameter and centre of mass to upper neck margin distance were associated with this phenotype. These findings suggest that selected aspects of proximal femoral geometry may be associated with a more severe fragility fracture phenotype beyond isolated hip/non-hip and even multiple non-hip fracture. Prospective studies are needed to determine whether HSA improves prediction of multiple fractures and whether its associations are independent of fracture sequence, BMD, falls and other determinants of fracture risk.

## Supplementary Information

Below is the link to the electronic supplementary material.

Supplementary Material 1
