# Supplementary material for: Hip Structural Analysis and Multiple Fragility Fractures: Evidence from a Large Cross-Sectional Clinical Cohort
Source: Calcif Tissue Int. 2026 Oct 18;117(1):163. doi: 10.1007/s00223-026-01625-4 (PMC13677640; doi:10.1007/s00223-026-01625-4)
Supplement: Supplementary file 1 [file 223_2026_1625_MOESM1_ESM.docx]

# 1. Supplementary Tables: Unadjusted vs Adjusted Odds Ratios (OR per 1 SD Increase)

For every HSA parameter and every fracture contrast, the unadjusted (univariate) model is shown alongside the fully adjusted model, so the influence of the FRAX/BMD covariates on each association can be assessed directly. Both models were fitted on the identical complete-case analytic sample for a given HSA parameter, and both were FDR-corrected (Benjamini-Hochberg) across the same 55 tests (44 multinomial + 11 binary) used in the main results document. Odds ratios are per 1 SD increase in the HSA parameter. Adjusted model covariates: age at scan, sex, BMI, current glucocorticoid use, rheumatoid arthritis, excess alcohol, current smoking, family history of fracture, and total femoral BMD (left hip in the primary analysis, right hip in the sensitivity analysis).

# 2. Primary Analysis — Left Hip HSA Parameters

## 2.1. Supplementary Table S1. Single Hip Fracture vs No Fracture

Supplementary Table S1. Single Hip Fracture vs No Fracture — Unadjusted vs Adjusted OR (per 1 SD Increase, 95% CI)

| **HSA Parameter** | **Unadjusted OR (95% CI)** | **Unadjusted FDR P** | **Sig. (Unadj.)** | **Adjusted OR (95% CI)** | **Adjusted FDR P** | **Sig. (Adj.)** |
| --- | --- | --- | --- | --- | --- | --- |
| Buckling Ratio | **2.19 (2.09–2.29)** | **<0.001** | ******* | 1.34 (0.96–1.87) | 0.40 |  |
| Centre of Mass to Upper Neck Margin (mm) | **1.53 (1.47–1.60)** | **<0.001** | ******* | 1.41 (1.01–1.97) | 0.33 |  |
| Cross-Sectional Area (mm²) | **0.52 (0.50–0.55)** | **<0.001** | ******* | 1.12 (0.80–1.56) | 1.00 |  |
| Cross-Sectional Moment of Inertia (mm⁴) | **0.91 (0.87–0.95)** | **<0.001** | ******* | 1.18 (0.85–1.65) | 1.00 |  |
| Femoral Head to Min-CSMI Section (mm) | 1.02 (0.98–1.07) | 0.49 |  | 1.08 (0.78–1.51) | 1.00 |  |
| Femoral Head to Shaft Axis (mm) | 1.01 (0.96–1.05) | 0.81 |  | 1.04 (0.75–1.45) | 1.00 |  |
| Femoral Shaft Angle (°) | **1.23 (1.10–1.38)** | **<0.001** | ******* | 1.13 (0.60–2.13) | 1.00 |  |
| Hip Axis Length (mm) | **1.33 (1.27–1.39)** | **<0.001** | ******* | 1.50 (1.07–2.09) | 0.17 |  |
| Neck Diameter (mm) | **1.36 (1.30–1.42)** | **<0.001** | ******* | 1.31 (0.94–1.82) | 0.49 |  |
| Neck-Shaft Angle (°) | **1.09 (1.05–1.14)** | **<0.001** | ******* | 1.01 (0.73–1.41) | 1.00 |  |
| Strength Index | **0.80 (0.76–0.83)** | **<0.001** | ******* | 0.87 (0.61–1.24) | 1.00 |  |
| *OR = odds ratio per 1 SD increase in the HSA parameter. Sig. (Unadj./Adj.): *** p<0.001, ** p<0.01, * p<0.05 (Benjamini-Hochberg FDR-adjusted, corrected jointly across the same 55 tests as the main results document).* | | | | | | |
| *Abbreviations: HAL = hip axis length; SI = strength index; CSMI = cross-sectional moment of inertia; CSA = cross-sectional area; NSA = neck-shaft angle; BR = buckling ratio.* | | | | | | |

## 2.2. Supplementary Table S2. Single Non-Hip Fracture vs No Fracture

Supplementary Table S2. Single Non-Hip Fracture vs No Fracture — Unadjusted vs Adjusted OR (per 1 SD Increase, 95% CI)

| **HSA Parameter** | **Unadjusted OR (95% CI)** | **Unadjusted FDR P** | **Sig. (Unadj.)** | **Adjusted OR (95% CI)** | **Adjusted FDR P** | **Sig. (Adj.)** |
| --- | --- | --- | --- | --- | --- | --- |
| Buckling Ratio | **1.34 (1.28–1.40)** | **<0.001** | ******* | 0.99 (0.78–1.25) | 1.00 |  |
| Centre of Mass to Upper Neck Margin (mm) | 1.04 (1.00–1.09) | 0.09 |  | 1.02 (0.81–1.28) | 1.00 |  |
| Cross-Sectional Area (mm²) | **0.74 (0.70–0.78)** | **<0.001** | ******* | 1.03 (0.82–1.30) | 1.00 |  |
| Cross-Sectional Moment of Inertia (mm⁴) | **0.86 (0.82–0.90)** | **<0.001** | ******* | 1.03 (0.81–1.29) | 1.00 |  |
| Femoral Head to Min-CSMI Section (mm) | **0.91 (0.87–0.95)** | **<0.001** | ******* | 0.99 (0.79–1.25) | 1.00 |  |
| Femoral Head to Shaft Axis (mm) | 0.99 (0.95–1.04) | 0.80 |  | 1.04 (0.83–1.31) | 1.00 |  |
| Femoral Shaft Angle (°) | 0.96 (0.85–1.08) | 0.60 |  | 0.93 (0.55–1.59) | 1.00 |  |
| Hip Axis Length (mm) | 0.96 (0.91–1.00) | 0.10 |  | 1.04 (0.82–1.31) | 1.00 |  |
| Neck Diameter (mm) | 0.99 (0.95–1.04) | 0.82 |  | 1.01 (0.80–1.28) | 1.00 |  |
| Neck-Shaft Angle (°) | 1.01 (0.96–1.05) | 0.81 |  | 0.98 (0.78–1.24) | 1.00 |  |
| Strength Index | **0.90 (0.86–0.94)** | **<0.001** | ******* | 1.02 (0.80–1.29) | 1.00 |  |
| *OR = odds ratio per 1 SD increase in the HSA parameter. Sig. (Unadj./Adj.): *** p<0.001, ** p<0.01, * p<0.05 (Benjamini-Hochberg FDR-adjusted, corrected jointly across the same 55 tests as the main results document).* | | | | | | |
| *Abbreviations: HAL = hip axis length; SI = strength index; CSMI = cross-sectional moment of inertia; CSA = cross-sectional area; NSA = neck-shaft angle; BR = buckling ratio.* | | | | | | |

## 2.3. Supplementary Table S3. Multiple Non-Hip Fractures vs No Fracture

Supplementary Table S3. Multiple Non-Hip Fractures vs No Fracture — Unadjusted vs Adjusted OR (per 1 SD Increase, 95% CI)

| **HSA Parameter** | **Unadjusted OR (95% CI)** | **Unadjusted FDR P** | **Sig. (Unadj.)** | **Adjusted OR (95% CI)** | **Adjusted FDR P** | **Sig. (Adj.)** |
| --- | --- | --- | --- | --- | --- | --- |
| Buckling Ratio | **1.44 (1.30–1.61)** | **<0.001** | ******* | 0.93 (0.30–2.89) | 1.00 |  |
| Centre of Mass to Upper Neck Margin (mm) | 1.02 (0.93–1.12) | 0.72 |  | 1.04 (0.48–2.24) | 1.00 |  |
| Cross-Sectional Area (mm²) | **0.63 (0.56–0.70)** | **<0.001** | ******* | 1.07 (0.36–3.13) | 1.00 |  |
| Cross-Sectional Moment of Inertia (mm⁴) | **0.77 (0.70–0.84)** | **<0.001** | ******* | 1.05 (0.47–2.36) | 1.00 |  |
| Femoral Head to Min-CSMI Section (mm) | **0.88 (0.80–0.96)** | **0.01** | ***** | 0.97 (0.45–2.09) | 1.00 |  |
| Femoral Head to Shaft Axis (mm) | 0.97 (0.89–1.07) | 0.69 |  | 1.08 (0.50–2.34) | 1.00 |  |
| Femoral Shaft Angle (°) | 0.90 (0.73–1.12) | 0.49 |  | 0.88 (0.16–4.89) | 1.00 |  |
| Hip Axis Length (mm) | 0.91 (0.83–1.00) | 0.08 |  | 1.06 (0.49–2.30) | 1.00 |  |
| Neck Diameter (mm) | 0.95 (0.86–1.04) | 0.38 |  | 1.01 (0.47–2.17) | 1.00 |  |
| Neck-Shaft Angle (°) | 0.98 (0.89–1.07) | 0.70 |  | 0.93 (0.43–2.01) | 1.00 |  |
| Strength Index | **0.83 (0.76–0.92)** | **<0.001** | ******* | 1.00 (0.44–2.29) | 1.00 |  |
| *OR = odds ratio per 1 SD increase in the HSA parameter. Sig. (Unadj./Adj.): *** p<0.001, ** p<0.01, * p<0.05 (Benjamini-Hochberg FDR-adjusted, corrected jointly across the same 55 tests as the main results document).* | | | | | | |
| *Abbreviations: HAL = hip axis length; SI = strength index; CSMI = cross-sectional moment of inertia; CSA = cross-sectional area; NSA = neck-shaft angle; BR = buckling ratio.* | | | | | | |

## 2.4. Supplementary Table S4. Multiple Fractures Including Hip vs No Fracture

Supplementary Table S4. Multiple Fractures Including Hip vs No Fracture — Unadjusted vs Adjusted OR (per 1 SD Increase, 95% CI)

| **HSA Parameter** | **Unadjusted OR (95% CI)** | **Unadjusted FDR P** | **Sig. (Unadj.)** | **Adjusted OR (95% CI)** | **Adjusted FDR P** | **Sig. (Adj.)** |
| --- | --- | --- | --- | --- | --- | --- |
| Buckling Ratio | **2.16 (2.00–2.34)** | **<0.001** | ******* | 1.14 (1.00–1.31) | 0.38 |  |
| Centre of Mass to Upper Neck Margin (mm) | **1.32 (1.22–1.44)** | **<0.001** | ******* | **1.35 (1.21–1.51)** | **<0.001** | ******* |
| Cross-Sectional Area (mm²) | **0.42 (0.37–0.47)** | **<0.001** | ******* | 1.18 (0.99–1.40) | 0.39 |  |
| Cross-Sectional Moment of Inertia (mm⁴) | **0.73 (0.65–0.81)** | **<0.001** | ******* | **1.25 (1.09–1.42)** | **0.01** | ***** |
| Femoral Head to Min-CSMI Section (mm) | 0.96 (0.88–1.05) | 0.49 |  | 1.06 (0.97–1.16) | 0.86 |  |
| Femoral Head to Shaft Axis (mm) | 0.96 (0.88–1.05) | 0.49 |  | 1.09 (0.99–1.20) | 0.39 |  |
| Femoral Shaft Angle (°) | 1.18 (0.97–1.42) | 0.15 |  | 1.08 (0.89–1.31) | 1.00 |  |
| Hip Axis Length (mm) | **1.11 (1.02–1.21)** | **0.04** | ***** | **1.44 (1.28–1.63)** | **<0.001** | ******* |
| Neck Diameter (mm) | **1.15 (1.05–1.25)** | **0.00** | ****** | **1.24 (1.11–1.38)** | **0.00** | ****** |
| Neck-Shaft Angle (°) | **1.14 (1.04–1.24)** | **0.01** | ***** | 1.05 (0.96–1.15) | 1.00 |  |
| Strength Index | **0.77 (0.70–0.85)** | **<0.001** | ******* | 0.86 (0.77–0.97) | 0.15 |  |
| *OR = odds ratio per 1 SD increase in the HSA parameter. Sig. (Unadj./Adj.): *** p<0.001, ** p<0.01, * p<0.05 (Benjamini-Hochberg FDR-adjusted, corrected jointly across the same 55 tests as the main results document).* | | | | | | |
| *Abbreviations: HAL = hip axis length; SI = strength index; CSMI = cross-sectional moment of inertia; CSA = cross-sectional area; NSA = neck-shaft angle; BR = buckling ratio.* | | | | | | |

## 2.5. Supplementary Table S5. Multiple Fractures Including Hip vs Single Hip Fracture

Supplementary Table S5. Multiple Fractures Including Hip vs Single Hip Fracture — Unadjusted vs Adjusted OR (per 1 SD Increase, 95% CI)

| **HSA Parameter** | **Unadjusted OR (95% CI)** | **Unadjusted FDR P** | **Sig. (Unadj.)** | **Adjusted OR (95% CI)** | **Adjusted FDR P** | **Sig. (Adj.)** |
| --- | --- | --- | --- | --- | --- | --- |
| Buckling Ratio | 0.98 (0.87–1.11) | 0.81 |  | 0.83 (0.68–1.03) | 0.40 |  |
| Centre of Mass to Upper Neck Margin (mm) | **0.84 (0.74–0.95)** | **0.01** | ****** | 0.96 (0.82–1.12) | 1.00 |  |
| Cross-Sectional Area (mm²) | **0.84 (0.74–0.95)** | **0.01** | ****** | 1.06 (0.85–1.33) | 1.00 |  |
| Cross-Sectional Moment of Inertia (mm⁴) | **0.82 (0.73–0.93)** | **0.01** | ****** | 1.06 (0.88–1.27) | 1.00 |  |
| Femoral Head to Min-CSMI Section (mm) | 0.94 (0.84–1.07) | 0.49 |  | 0.97 (0.85–1.10) | 1.00 |  |
| Femoral Head to Shaft Axis (mm) | 0.96 (0.85–1.08) | 0.60 |  | 1.04 (0.91–1.19) | 1.00 |  |
| Femoral Shaft Angle (°) | 0.95 (0.71–1.26) | 0.80 |  | 0.98 (0.72–1.33) | 1.00 |  |
| Hip Axis Length (mm) | **0.81 (0.71–0.92)** | **0.00** | ****** | 0.94 (0.80–1.11) | 1.00 |  |
| Neck Diameter (mm) | **0.82 (0.73–0.93)** | **0.00** | ****** | 0.95 (0.81–1.12) | 1.00 |  |
| Neck-Shaft Angle (°) | 1.04 (0.92–1.17) | 0.69 |  | 1.04 (0.91–1.18) | 1.00 |  |
| Strength Index | 0.97 (0.86–1.10) | 0.73 |  | 1.00 (0.86–1.16) | 1.00 |  |
| *OR = odds ratio per 1 SD increase in the HSA parameter. Sig. (Unadj./Adj.): *** p<0.001, ** p<0.01, * p<0.05 (Benjamini-Hochberg FDR-adjusted, corrected jointly across the same 55 tests as the main results document).* | | | | | | |
| *Abbreviations: HAL = hip axis length; SI = strength index; CSMI = cross-sectional moment of inertia; CSA = cross-sectional area; NSA = neck-shaft angle; BR = buckling ratio.* | | | | | | |

# 3. Sensitivity Analysis — Right Hip HSA Parameters

## 3.1. Supplementary Table S1R. Single Hip Fracture vs No Fracture

Supplementary Table S1R. Single Hip Fracture vs No Fracture (Right Hip) — Unadjusted vs Adjusted OR (per 1 SD Increase, 95% CI)

| **HSA Parameter** | **Unadjusted OR (95% CI)** | **Unadjusted FDR P** | **Sig. (Unadj.)** | **Adjusted OR (95% CI)** | **Adjusted FDR P** | **Sig. (Adj.)** |
| --- | --- | --- | --- | --- | --- | --- |
| Buckling Ratio | **2.20 (2.10–2.30)** | **<0.001** | ******* | 1.32 (0.90–1.95) | 0.57 |  |
| Centre of Mass to Upper Neck Margin (mm) | **1.62 (1.55–1.69)** | **<0.001** | ******* | 1.55 (1.05–2.29) | 0.20 |  |
| Cross-Sectional Area (mm²) | **0.54 (0.51–0.56)** | **<0.001** | ******* | 1.21 (0.82–1.78) | 0.82 |  |
| Cross-Sectional Moment of Inertia (mm⁴) | **0.93 (0.89–0.97)** | **0.00** | ****** | 1.27 (0.86–1.87) | 0.67 |  |
| Femoral Head to Min-CSMI Section (mm) | **1.07 (1.02–1.12)** | **0.01** | ****** | 1.14 (0.77–1.68) | 0.99 |  |
| Femoral Head to Shaft Axis (mm) | 1.00 (0.96–1.05) | 0.96 |  | 1.05 (0.71–1.55) | 0.99 |  |
| Femoral Shaft Angle (°) | **1.39 (1.25–1.55)** | **<0.001** | ******* | 1.24 (0.58–2.63) | 0.99 |  |
| Hip Axis Length (mm) | **1.36 (1.30–1.42)** | **<0.001** | ******* | 1.54 (1.04–2.27) | 0.20 |  |
| Neck Diameter (mm) | **1.38 (1.32–1.44)** | **<0.001** | ******* | 1.33 (0.90–1.96) | 0.57 |  |
| Neck-Shaft Angle (°) | **1.08 (1.03–1.13)** | **0.00** | ****** | 0.99 (0.67–1.46) | 0.99 |  |
| Strength Index | **0.78 (0.75–0.82)** | **<0.001** | ******* | 0.82 (0.55–1.23) | 0.82 |  |
| *OR = odds ratio per 1 SD increase in the HSA parameter. Sig. (Unadj./Adj.): *** p<0.001, ** p<0.01, * p<0.05 (Benjamini-Hochberg FDR-adjusted, corrected jointly across the same 55 right-hip tests as the main results document).* | | | | | | |
| *Abbreviations: HAL = hip axis length; SI = strength index; CSMI = cross-sectional moment of inertia; CSA = cross-sectional area; NSA = neck-shaft angle; BR = buckling ratio.* | | | | | | |

## 3.2. Supplementary Table S2R. Single Non-Hip Fracture vs No Fracture

Supplementary Table S2R. Single Non-Hip Fracture vs No Fracture (Right Hip) — Unadjusted vs Adjusted OR (per 1 SD Increase, 95% CI)

| **HSA Parameter** | **Unadjusted OR (95% CI)** | **Unadjusted FDR P** | **Sig. (Unadj.)** | **Adjusted OR (95% CI)** | **Adjusted FDR P** | **Sig. (Adj.)** |
| --- | --- | --- | --- | --- | --- | --- |
| Buckling Ratio | **1.33 (1.27–1.39)** | **<0.001** | ******* | 0.97 (0.77–1.22) | 0.99 |  |
| Centre of Mass to Upper Neck Margin (mm) | **1.06 (1.01–1.10)** | **0.03** | ***** | 1.03 (0.82–1.30) | 0.99 |  |
| Cross-Sectional Area (mm²) | **0.74 (0.70–0.78)** | **<0.001** | ******* | 1.02 (0.81–1.29) | 0.99 |  |
| Cross-Sectional Moment of Inertia (mm⁴) | **0.86 (0.82–0.90)** | **<0.001** | ******* | 1.01 (0.80–1.27) | 0.99 |  |
| Femoral Head to Min-CSMI Section (mm) | **0.90 (0.86–0.94)** | **<0.001** | ******* | 0.97 (0.77–1.22) | 0.99 |  |
| Femoral Head to Shaft Axis (mm) | 0.99 (0.95–1.04) | 0.85 |  | 1.05 (0.84–1.32) | 0.99 |  |
| Femoral Shaft Angle (°) | 1.01 (0.91–1.13) | 0.88 |  | 0.99 (0.58–1.68) | 0.99 |  |
| Hip Axis Length (mm) | 0.97 (0.92–1.01) | 0.18 |  | 1.04 (0.83–1.31) | 0.99 |  |
| Neck Diameter (mm) | 1.00 (0.95–1.04) | 0.88 |  | 1.00 (0.80–1.26) | 0.99 |  |
| Neck-Shaft Angle (°) | 1.00 (0.96–1.05) | 0.99 |  | 0.97 (0.77–1.23) | 0.99 |  |
| Strength Index | **0.90 (0.86–0.94)** | **<0.001** | ******* | 1.01 (0.80–1.27) | 0.99 |  |
| *OR = odds ratio per 1 SD increase in the HSA parameter. Sig. (Unadj./Adj.): *** p<0.001, ** p<0.01, * p<0.05 (Benjamini-Hochberg FDR-adjusted, corrected jointly across the same 55 right-hip tests as the main results document).* | | | | | | |
| *Abbreviations: HAL = hip axis length; SI = strength index; CSMI = cross-sectional moment of inertia; CSA = cross-sectional area; NSA = neck-shaft angle; BR = buckling ratio.* | | | | | | |

## 3.3. Supplementary Table S3R. Multiple Non-Hip Fractures vs No Fracture

Supplementary Table S3R. Multiple Non-Hip Fractures vs No Fracture (Right Hip) — Unadjusted vs Adjusted OR (per 1 SD Increase, 95% CI)

| **HSA Parameter** | **Unadjusted OR (95% CI)** | **Unadjusted FDR P** | **Sig. (Unadj.)** | **Adjusted OR (95% CI)** | **Adjusted FDR P** | **Sig. (Adj.)** |
| --- | --- | --- | --- | --- | --- | --- |
| Buckling Ratio | **1.46 (1.31–1.63)** | **<0.001** | ******* | 0.93 (0.30–2.86) | 0.99 |  |
| Centre of Mass to Upper Neck Margin (mm) | 1.03 (0.93–1.13) | 0.69 |  | 1.03 (0.48–2.20) | 0.99 |  |
| Cross-Sectional Area (mm²) | **0.62 (0.55–0.69)** | **<0.001** | ******* | 1.03 (0.35–2.98) | 0.99 |  |
| Cross-Sectional Moment of Inertia (mm⁴) | **0.76 (0.70–0.84)** | **<0.001** | ******* | 1.04 (0.47–2.31) | 0.99 |  |
| Femoral Head to Min-CSMI Section (mm) | **0.87 (0.79–0.95)** | **0.00** | ****** | 0.97 (0.46–2.03) | 0.99 |  |
| Femoral Head to Shaft Axis (mm) | 0.96 (0.88–1.06) | 0.53 |  | 1.08 (0.51–2.30) | 0.99 |  |
| Femoral Shaft Angle (°) | 1.00 (0.81–1.23) | 0.99 |  | 0.97 (0.21–4.54) | 0.99 |  |
| Hip Axis Length (mm) | 0.92 (0.84–1.01) | 0.10 |  | 1.06 (0.50–2.25) | 0.99 |  |
| Neck Diameter (mm) | 0.94 (0.86–1.03) | 0.27 |  | 1.00 (0.47–2.11) | 0.99 |  |
| Neck-Shaft Angle (°) | 1.01 (0.93–1.11) | 0.85 |  | 0.97 (0.46–2.05) | 0.99 |  |
| Strength Index | **0.84 (0.77–0.93)** | **0.00** | ****** | 1.01 (0.45–2.28) | 0.99 |  |
| *OR = odds ratio per 1 SD increase in the HSA parameter. Sig. (Unadj./Adj.): *** p<0.001, ** p<0.01, * p<0.05 (Benjamini-Hochberg FDR-adjusted, corrected jointly across the same 55 right-hip tests as the main results document).* | | | | | | |
| *Abbreviations: HAL = hip axis length; SI = strength index; CSMI = cross-sectional moment of inertia; CSA = cross-sectional area; NSA = neck-shaft angle; BR = buckling ratio.* | | | | | | |

## 3.4. Supplementary Table S4R. Multiple Fractures Including Hip vs No Fracture

Supplementary Table S4R. Multiple Fractures Including Hip vs No Fracture (Right Hip) — Unadjusted vs Adjusted OR (per 1 SD Increase, 95% CI)

| **HSA Parameter** | **Unadjusted OR (95% CI)** | **Unadjusted FDR P** | **Sig. (Unadj.)** | **Adjusted OR (95% CI)** | **Adjusted FDR P** | **Sig. (Adj.)** |
| --- | --- | --- | --- | --- | --- | --- |
| Buckling Ratio | **2.28 (2.11–2.46)** | **<0.001** | ******* | 1.18 (1.03–1.35) | 0.17 |  |
| Centre of Mass to Upper Neck Margin (mm) | **1.34 (1.23–1.46)** | **<0.001** | ******* | **1.30 (1.17–1.45)** | **<0.001** | ******* |
| Cross-Sectional Area (mm²) | **0.40 (0.36–0.45)** | **<0.001** | ******* | 1.09 (0.92–1.31) | 0.82 |  |
| Cross-Sectional Moment of Inertia (mm⁴) | **0.70 (0.63–0.78)** | **<0.001** | ******* | 1.14 (0.99–1.30) | 0.37 |  |
| Femoral Head to Min-CSMI Section (mm) | 0.93 (0.85–1.02) | 0.18 |  | 1.04 (0.95–1.14) | 0.95 |  |
| Femoral Head to Shaft Axis (mm) | 0.92 (0.84–1.00) | 0.09 |  | 1.07 (0.97–1.17) | 0.63 |  |
| Femoral Shaft Angle (°) | 1.17 (0.97–1.41) | 0.15 |  | 1.04 (0.86–1.26) | 0.99 |  |
| Hip Axis Length (mm) | **1.12 (1.02–1.22)** | **0.02** | ***** | **1.37 (1.22–1.54)** | **<0.001** | ******* |
| Neck Diameter (mm) | **1.17 (1.08–1.28)** | **<0.001** | ******* | **1.20 (1.07–1.33)** | **0.02** | ***** |
| Neck-Shaft Angle (°) | **1.12 (1.02–1.23)** | **0.02** | ***** | 1.02 (0.93–1.12) | 0.99 |  |
| Strength Index | **0.69 (0.62–0.77)** | **<0.001** | ******* | **0.82 (0.73–0.92)** | **0.02** | ***** |
| *OR = odds ratio per 1 SD increase in the HSA parameter. Sig. (Unadj./Adj.): *** p<0.001, ** p<0.01, * p<0.05 (Benjamini-Hochberg FDR-adjusted, corrected jointly across the same 55 right-hip tests as the main results document).* | | | | | | |
| *Abbreviations: HAL = hip axis length; SI = strength index; CSMI = cross-sectional moment of inertia; CSA = cross-sectional area; NSA = neck-shaft angle; BR = buckling ratio.* | | | | | | |

## 3.5. Supplementary Table S5R. Multiple Fractures Including Hip vs Single Hip Fracture

Supplementary Table S5R. Multiple Fractures Including Hip vs Single Hip Fracture (Right Hip) — Unadjusted vs Adjusted OR (per 1 SD Increase, 95% CI)

| **HSA Parameter** | **Unadjusted OR (95% CI)** | **Unadjusted FDR P** | **Sig. (Unadj.)** | **Adjusted OR (95% CI)** | **Adjusted FDR P** | **Sig. (Adj.)** |
| --- | --- | --- | --- | --- | --- | --- |
| Buckling Ratio | 1.05 (0.93–1.19) | 0.51 |  | 0.88 (0.72–1.08) | 0.66 |  |
| Centre of Mass to Upper Neck Margin (mm) | **0.79 (0.70–0.90)** | **<0.001** | ******* | 0.83 (0.71–0.97) | 0.17 |  |
| Cross-Sectional Area (mm²) | **0.79 (0.70–0.90)** | **<0.001** | ******* | 0.90 (0.72–1.11) | 0.82 |  |
| Cross-Sectional Moment of Inertia (mm⁴) | **0.78 (0.68–0.88)** | **<0.001** | ******* | 0.89 (0.74–1.06) | 0.63 |  |
| Femoral Head to Min-CSMI Section (mm) | 0.88 (0.78–1.00) | 0.07 |  | 0.90 (0.79–1.02) | 0.50 |  |
| Femoral Head to Shaft Axis (mm) | 0.92 (0.82–1.04) | 0.26 |  | 1.01 (0.89–1.16) | 0.99 |  |
| Femoral Shaft Angle (°) | 0.81 (0.61–1.06) | 0.18 |  | 0.81 (0.59–1.08) | 0.57 |  |
| Hip Axis Length (mm) | **0.80 (0.71–0.91)** | **0.00** | ****** | 0.88 (0.75–1.05) | 0.57 |  |
| Neck Diameter (mm) | **0.83 (0.73–0.94)** | **0.01** | ****** | 0.88 (0.75–1.03) | 0.50 |  |
| Neck-Shaft Angle (°) | 1.03 (0.92–1.17) | 0.68 |  | 1.06 (0.93–1.20) | 0.94 |  |
| Strength Index | 0.90 (0.79–1.02) | 0.14 |  | 1.00 (0.86–1.15) | 0.99 |  |
| *OR = odds ratio per 1 SD increase in the HSA parameter. Sig. (Unadj./Adj.): *** p<0.001, ** p<0.01, * p<0.05 (Benjamini-Hochberg FDR-adjusted, corrected jointly across the same 55 right-hip tests as the main results document).* | | | | | | |
| *Abbreviations: HAL = hip axis length; SI = strength index; CSMI = cross-sectional moment of inertia; CSA = cross-sectional area; NSA = neck-shaft angle; BR = buckling ratio.* | | | | | | |
